# Crystal structures of cristobalite-type and coesite-type PON redetermined on the basis of single-crystal X-ray diffraction data

**DOI:** 10.1107/S205698901501899X

**Published:** 2015-10-14

**Authors:** Maxim Bykov, Elena Bykova, Vadim Dyadkin, Dominik Baumann, Wolfgang Schnick, Leonid Dubrovinsky, Natalia Dubrovinskaia

**Affiliations:** a Bayerisches Geoinstitut, University of Bayreuth, 95440 Bayreuth, Germany; b ESRF, 38043 Grenoble, France; c Department of Chemistry, Ludwig Maximilian University, 81377 Munich, Germany; d Material Physics and Technology at Extreme Conditions, Laboratory of Crystallography, University of Bayreuth, 95440 Bayreuth, Germany

**Keywords:** crystal structure, phospho­rus oxonitride, silica analogues, redetermination

## Abstract

Hitherto, phospho­rus oxonitride (PON) could not be obtained in the form of single crystals and only powder diffraction experiments were feasible for structure studies. In the present work we have synthesized two polymorphs of phospho­rus oxonitride, cristobalite-type (*cri*-PON) and coesite-type (*coe*-PON), in the form of single crystals and reinvestigated their crystal structures by means of in house and synchrotron single-crystal X-ray diffraction. The crystal structures of *cri*-PON and *coe*-PON are built from PO_2_N_2_ tetra­hedral units, each with a statistical distribution of oxygen and nitro­gen atoms. The crystal structure of the *coe*-PON phase has the space group *C*2*/c* with seven atomic sites in the asymmetric unit [two P and three (N,O) sites on general positions, one (N,O) site on an inversion centre and one (N,O) site on a twofold rotation axis], while the *cri*-PON phase possesses tetra­gonal *I*-42*d* symmetry with two independent atoms in the asymmetric unit [the P atom on a fourfold inversion axis and the (N,O) site on a twofold rotation axis]. In comparison with previous structure determinations from powder data, all atoms were refined with anisotropic displacement parameters, leading to higher precision in terms of bond lengths and angles.

## Abstract 2

The crystal structures of two phospho­rus oxonitride polymorphs (cristobalite- and coesite-type) were redetermined by means of single-crystal X-ray diffraction data.

## Chemical context   

The pseudo-binary system P_3_N_5_/P_2_O_5_ has been investigated intensively because the properties of related ceramic materials are promising for industrial applications. A mid-member of this system is phospho­rus oxonitride (PON), whose chemical stability is essential for its use as an insulator or for fireproofing. This compound has attracted significant attention as a ternary base compound of electrolytes for rechargeable thin-film Li/Li-ion batteries. Phospho­rus oxonitride is an isoelectronic analogue of silica (SiO_2_) with the charge-balanced substitution P^5+^ + N^3−^ = Si^4+^ + O^2−^. The crystal structures of the polymorphic forms of SiO_2_ and PON are built of tetra­hedral SiO_4_ and PO_2_N_2_ units, respectively. At present, five modifications of PON have been identified. Four of them are isostructural to known silica polymorphs, *viz.* α-quartz- (Léger *et al.*, 1999 ▸), β-cristobalite- (Léger *et al.*, 2001 ▸), moganite- (Chateau *et al.*, 1999 ▸) and coesite-type (Baumann *et al.*, 2015 ▸). The fifth one, δ-PON, has a structure type different from any of the silica modifications (Baumann *et al.*, 2012 ▸). A rich variety of polymorphs is a result of the many ways in which the tetra­hedra can be linked to form corner-sharing networks. Most of the phases in the P_3_N_5_/P_2_O_5_ system are usually obtained either in an amorphous state or in the form of powders consisting of very small crystallites. We succeeded in synthesizing single crystals of pure cristobalite- (*cri*) and coesite-type (*coe*) PON of a size suitable for single-crystal X-ray diffraction and report here the results of the structure refinements.

## Structural commentary   

**Figure 1:**
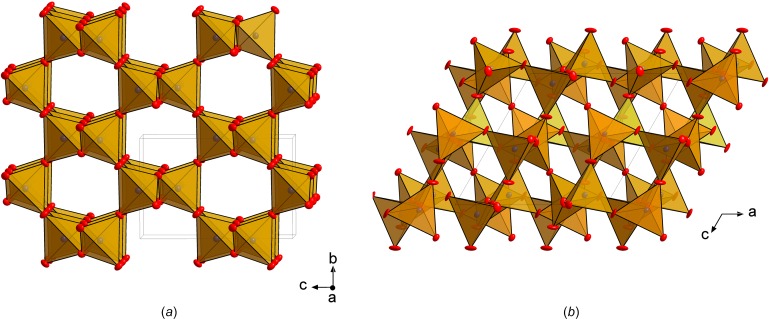
Crystal structures of *cri*-PON (*a*) and *coe*-PON (*b*) shown in polyhedral representation. Displacement parameters are drawn at the 50% probability level. Mixed (N,O) sites are shown in red; P atoms are shown in brown.

The structure of *cri*-PON (Fig. 1 ▸ *a*) can be derived from that of β-cristobalite by tilting each PO_2_N_2_ tetra­hedron about the 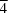 axes alternately clockwise and anti­clockwise. This leads to the lowering of symmetry from *Fd* 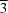 *m* to *I* 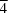
2*d*, however, the topology remains the same. The length of the P—(O,N) bond in *cri*-PON is 1.5796 (10) Å, which is in a good agreement with the average of expected P—N (1.626 Å) and P—O (1.537 Å) distances (Huminicki & Hawthorne, 2002 ▸). All P—(O,N) distances within the PO_2_N_2_ units are equal, but there is a noticeable (O,N)—P—(O,N) angle variation between 107.86 (2) and 112.73 (5)° due to the compression of the tetra­hedra along the *c-*axis direction.

The structure of *coe*-PON (Fig. 1 ▸ *b*) is isotypic with coesite (SiO_2_) (Angel *et al.*, 2003 ▸). The framework of *coe*-PON is constructed of four-member rings comprised of corner-sharing PO_2_N_2_ tetra­hedra. These rings are linked in such a manner that crankshaft-like chains are formed. The average P—(O,N) distance in *coe*-PON (1.572 Å) is slightly shorter than that of 1.581 Å reported by Baumann *et al.* (2015 ▸) likely due to the difference in temperatures at which the experiments were conducted. The tetra­hedra are irregularly distorted, with P—(O,N) distances varying between 1.5530 (9) and 1.588 (3) Å, and (O,N)—P—(O,N) angles between 106.79 (19) and 112.0 (2)°.

In comparison with the refinements from powder diffraction data (Léger *et al.*, 2001 ▸; Baumann *et al.*, 2015 ▸), single-crystal diffraction data revealed a detailed electron density map, which allowed us in addition to a substitutional O-N disorder, to detect a possible positional disorder (for details see *Refinement* section), which may affect physical properties of *coe*-PON.

## Synthesis and crystallisation   

Cristobalite-type PON was synthesized from phospho­ric tri­amide by a two-step condensation process. POCl_3_ (99%, Sigma Aldrich) was reacted with liquid NH_3_ (5.0, Air Liquide) to yield a mixture of PO(NH_2_)_3_ and NH_4_Cl, which was subsequently heated to 893 K for 5 h in a stream of dry ammonia. The amorphous reaction product was crystallized at 1023 K for 7 d in an evacuated fused silica ampoule, yielding pure cristobalite-type PON. Coesite-type PON was obtained by high-pressure/high-temperature reaction of *cri*-PON in a modified Walker-type multi-anvil apparatus. The starting material was tightly packed in a *h*-BN capsule, which was centered in a MgO:Cr octa­hedron (Ceramic Substrates & Components, Isle of Wight, UK) with an edge length of 10 mm. The latter was subsequently compressed between eight truncated tungsten carbide cubes (5 mm truncation edge length, Hawedia, Marklkofen, Germany) using a 1000 t hydraulic press (Voggenreiter, Mainleus, Germany). The sample was compressed to 15.5 GPa, the temperature raised to 1573 K within 15 min and held constant for 60 min. The sample was cooled by turning off the heating, decompressed and mechanically isolated.

## Refinement   

Crystal data, data collection and structure refinement details are summarized in Table 1 ▸. Structure refinements of both *coe*-PON and *cri*-PON were performed using occupancies of oxygen and nitro­gen atoms fixed to 0.5 for each site. As a result of the very similar scattering powers of N and O atoms, an attempt to refine the occupancies resulted in unreliable values with large standard uncertainties. The *cri*-PON crystal was twinned by inversion with an equal amount of the two twin domains. The refinement of the *coe*-PON structure revealed a residual electron density peak of 1.41 e^−^·Å^−3^ at a distance 1.22 Å from atom P2 and 1.50, 1.65 and 1.65 Å from atoms O1, O2 and O5, respectively. This density may be explained by a static disorder of the P2 atom between two positions. The disorder is, however, too weak to give additional reliable residual density peaks for the assignments of oxygen and nitro­gen atoms.

## Supplementary Material

Crystal structure: contains datablock(s) coe-PON, New_Global_Publ_Block, cri-PON. DOI: 10.1107/S205698901501899X/wm5203sup1.cif

Structure factors: contains datablock(s) cri-PON. DOI: 10.1107/S205698901501899X/wm5203cri-PONsup2.hkl

Structure factors: contains datablock(s) coe-PON. DOI: 10.1107/S205698901501899X/wm5203coe-PONsup3.hkl

CCDC references: 1430221, 1430220

Additional supporting information:  crystallographic information; 3D view; checkCIF report

## Figures and Tables

**Table 1:** Experimental details

	*cri*-PON	*coe*-PON
Crystal data		
Chemical formula	PON	PON
*M*_r_	60.98	60.98
Crystal system, space group	Tetragonal, *I* 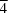 2*d*	Monoclinic, *C*2/*c*
Temperature (K)	293	100
*a*, *b*, *c* (Å)	4.6135 (2), 4.6135 (2), 6.9991 (5)	6.9464 (6), 12.0340 (4), 6.9463 (5)
α, β, γ (°)	90, 90, 90	90, 119.914 (10), 90
*V* (Å^3^)	148.97 (2)	503.30 (7)
*Z*	4	16
Radiation type	Mo *K*α	Synchrotron, λ = 0.69428 Å
μ (mm^−1^)	1.24	1.35
Crystal size (mm)	0.02 × 0.02 × 0.02	0.02 × 0.02 × 0.02

Data collection		
Diffractometer	Bruker SMART APEX CCD	PILATUS@SNBL
Absorption correction	Multi-scan (*CrysAlis PRO*; Agilent, 2014 ▸)	Multi-scan (*CrysAlis PRO*; Agilent, 2014 ▸)
*T*_min_, *T*_max_	0.791, 1.000	0.949, 1.000
No. of measured, independent and observed [*I* > 2σ(*I*)] reflections	445, 92, 92	2415, 535, 469
*R*_int_	0.016	0.038
(sin θ/λ)_max_ (Å^−1^)	0.666	0.640

Refinement		
*R*[*F* ^2^ > 2σ(*F* ^2^)], *wR*(*F* ^2^), *S*	0.016, 0.043, 1.45	0.037, 0.102, 1.05
No. of reflections	92	535
No. of parameters	8	57
Δρ_max_, Δρ_min_ (e Å^−3^)	0.21, −0.28	1.41, −0.54
Absolute structure	Refined as a perfect inversion twin.	–
Absolute structure parameter	0.5	–

Computer programs: *CrysAlis PRO* (Agilent, 2014 ▸), *SHELXS* (Sheldrick, 2008 ▸), *SHELXL2014/7* (Sheldrick, 2015 ▸), *DIAMOND* (Brandenburg, 2006 ▸) and *publCIF* (Westrip, 2010 ▸).

## supplementary crystallographic information

### (cri-PON) Phosphorus oxonitride Crystal data

**Table:**

NOP	*D*_x_ = 2.719 Mg m^−^^3^
*M**_r_* = 60.98	Mo *K*α radiation, λ = 0.71069 Å
Tetragonal, *I*42*d*	Cell parameters from 431 reflections
*a* = 4.6135 (2) Å	θ = 5.3–28.2°
*c* = 6.9991 (5) Å	µ = 1.24 mm^−^^1^
*V* = 148.97 (2) Å^3^	*T* = 293 K
*Z* = 4	Prism, colourless
*F*(000) = 120	0.02 × 0.02 × 0.02 mm

### (cri-PON) Phosphorus oxonitride Data collection

**Table:**

Three-circle diffractometer	92 independent reflections
Radiation source: rotating-anode X-ray tube, Rigaku Rotor Flex FR-D	92 reflections with *I* > 2σ(*I*)
Detector resolution: 16.6 pixels mm^-1^	*R*_int_ = 0.016
ω scans	θ_max_ = 28.3°, θ_min_ = 5.3°
Absorption correction: multi-scan (*CrysAlis PRO*; Agilent, 2014)	*h* = −5→5
*T*_min_ = 0.791, *T*_max_ = 1.000	*k* = −5→6
445 measured reflections	*l* = −5→9

### (cri-PON) Phosphorus oxonitride Refinement

**Table:**

Refinement on *F*^2^	0 restraints
Least-squares matrix: full	*w* = 1/[σ^2^(*F*_o_^2^) + (0.0229*P*)^2^ + 0.0508*P*] where *P* = (*F*_o_^2^ + 2*F*_c_^2^)/3
*R*[*F*^2^ > 2σ(*F*^2^)] = 0.016	(Δ/σ)_max_ < 0.001
*wR*(*F*^2^) = 0.043	Δρ_max_ = 0.21 e Å^−^^3^
*S* = 1.45	Δρ_min_ = −0.28 e Å^−^^3^
92 reflections	Absolute structure: Refined as a perfect inversion twin.
8 parameters	Absolute structure parameter: 0.5

### (cri-PON) Phosphorus oxonitride Special details

**Table:**

Geometry. All e.s.d.'s (except the e.s.d. in the dihedral angle between two l.s. planes) are estimated using the full covariance matrix. The cell e.s.d.'s are taken into account individually in the estimation of e.s.d.'s in distances, angles and torsion angles; correlations between e.s.d.'s in cell parameters are only used when they are defined by crystal symmetry. An approximate (isotropic) treatment of cell e.s.d.'s is used for estimating e.s.d.'s involving l.s. planes.
Refinement. Refined as a 2-component perfect inversion twin.

### (cri-PON) Phosphorus oxonitride Fractional atomic coordinates and isotropic or equivalent isotropic displacement parameters (Å^2^)

**Table:**

	*x*	*y*	*z*	*U*_iso_*/*U*_eq_	Occ. (<1)
P	0.5000	0.5000	0.0000	0.0106 (3)	
N	0.3630 (5)	0.2500	0.1250	0.0155 (5)	0.5
O	0.3630 (5)	0.2500	0.1250	0.0155 (5)	0.5

### (cri-PON) Phosphorus oxonitride Atomic displacement parameters (Å^2^)

**Table:**

	*U*^11^	*U*^22^	*U*^33^	*U*^12^	*U*^13^	*U*^23^
P	0.0112 (4)	0.0112 (4)	0.0094 (4)	0.000	0.000	0.000
N	0.0151 (11)	0.0149 (12)	0.0165 (9)	0.000	0.000	0.0065 (10)
O	0.0151 (11)	0.0149 (12)	0.0165 (9)	0.000	0.000	0.0065 (10)

### (cri-PON) Phosphorus oxonitride Geometric parameters (Å, º)

**Table:**

P—O^i^	1.5796 (10)	P—O^iii^	1.5796 (10)
P—N^i^	1.5796 (10)	P—N^iii^	1.5796 (10)
P—O^ii^	1.5796 (10)	P—N	1.5796 (10)
P—N^ii^	1.5796 (10)	N—P^iv^	1.5796 (10)
			
O^i^—P—N^i^	0.0	N^i^—P—N^iii^	107.86 (2)
O^i^—P—O^ii^	107.86 (2)	O^ii^—P—N^iii^	112.7
N^i^—P—O^ii^	107.86 (2)	N^ii^—P—N^iii^	112.73 (5)
O^i^—P—N^ii^	107.9	O^iii^—P—N^iii^	0.0
N^i^—P—N^ii^	107.86 (2)	O^i^—P—N	112.7
O^ii^—P—N^ii^	0.0	N^i^—P—N	112.73 (5)
O^i^—P—O^iii^	107.86 (2)	O^ii^—P—N	107.9
N^i^—P—O^iii^	107.86 (2)	N^ii^—P—N	107.86 (2)
O^ii^—P—O^iii^	112.73 (5)	O^iii^—P—N	107.9
N^ii^—P—O^iii^	112.73 (5)	N^iii^—P—N	107.86 (2)
O^i^—P—N^iii^	107.9	P—N—P^iv^	132.83 (16)

Symmetry codes: (i) −*x*+1, −*y*+1, *z*; (ii) *y*, −*x*+1, −*z*; (iii) −*y*+1, *x*, −*z*; (iv) *x*, −*y*+1/2, −*z*+1/4.

### (coe-PON) Phosphorus oxonitride Crystal data

**Table:**

NOP	*F*(000) = 480
*M**_r_* = 60.98	*D*_x_ = 3.219 Mg m^−^^3^
Monoclinic, *C*2/*c*	Synchrotron radiation, λ = 0.69428 Å
*a* = 6.9464 (6) Å	Cell parameters from 1202 reflections
*b* = 12.0340 (4) Å	θ = 3.3–26.3°
*c* = 6.9463 (5) Å	µ = 1.35 mm^−^^1^
β = 119.914 (10)°	*T* = 100 K
*V* = 503.30 (7) Å^3^	Prism, colourless
*Z* = 16	0.02 × 0.02 × 0.02 mm

### (coe-PON) Phosphorus oxonitride Data collection

**Table:**

PILATUS@SNBL diffractometer	535 independent reflections
Radiation source: Beamline BM1A, SNBL ESRF, Grenoble, France	469 reflections with *I* > 2σ(*I*)
Detector resolution: 5.8 pixels mm^-1^	*R*_int_ = 0.038
φ scans	θ_max_ = 26.4°, θ_min_ = 3.3°
Absorption correction: multi-scan (*CrysAlis PRO*; Agilent, 2014)	*h* = −8→8
*T*_min_ = 0.949, *T*_max_ = 1.000	*k* = −15→15
2415 measured reflections	*l* = −8→8

### (coe-PON) Phosphorus oxonitride Refinement

**Table:**

Refinement on *F*^2^	57 parameters
Least-squares matrix: full	0 restraints
*R*[*F*^2^ > 2σ(*F*^2^)] = 0.037	*w* = 1/[σ^2^(*F*_o_^2^) + (0.054*P*)^2^ + 4.3556*P*] where *P* = (*F*_o_^2^ + 2*F*_c_^2^)/3
*wR*(*F*^2^) = 0.102	(Δ/σ)_max_ < 0.001
*S* = 1.05	Δρ_max_ = 1.41 e Å^−^^3^
535 reflections	Δρ_min_ = −0.54 e Å^−^^3^

### (coe-PON) Phosphorus oxonitride Special details

**Table:**

Geometry. All e.s.d.'s (except the e.s.d. in the dihedral angle between two l.s. planes) are estimated using the full covariance matrix. The cell e.s.d.'s are taken into account individually in the estimation of e.s.d.'s in distances, angles and torsion angles; correlations between e.s.d.'s in cell parameters are only used when they are defined by crystal symmetry. An approximate (isotropic) treatment of cell e.s.d.'s is used for estimating e.s.d.'s involving l.s. planes.

### (coe-PON) Phosphorus oxonitride Fractional atomic coordinates and isotropic or equivalent isotropic displacement parameters (Å^2^)

**Table:**

	*x*	*y*	*z*	*U*_iso_*/*U*_eq_	Occ. (<1)
P1	0.28266 (16)	0.09026 (8)	0.04006 (16)	0.0067 (4)	
P2	0.31812 (17)	0.35749 (7)	0.42525 (17)	0.0084 (4)	
N1	0.2117 (5)	0.4603 (2)	0.4818 (6)	0.0148 (8)	0.5
O1	0.2117 (5)	0.4603 (2)	0.4818 (6)	0.0148 (8)	0.5
N2	0.2500	0.2500	0.5000	0.0116 (10)	0.5
O2	0.2500	0.2500	0.5000	0.0116 (10)	0.5
N3	0.2322 (6)	0.3532 (3)	0.1704 (5)	0.0188 (8)	0.5
O3	0.2322 (6)	0.3532 (3)	0.1704 (5)	0.0188 (8)	0.5
N4	0.5000	0.1336 (3)	0.2500	0.0110 (10)	0.5
O4	0.5000	0.1336 (3)	0.2500	0.0110 (10)	0.5
N5	0.0792 (5)	0.1273 (3)	0.0656 (6)	0.0186 (8)	0.5
O5	0.0792 (5)	0.1273 (3)	0.0656 (6)	0.0186 (8)	0.5

### (coe-PON) Phosphorus oxonitride Atomic displacement parameters (Å^2^)

**Table:**

	*U*^11^	*U*^22^	*U*^33^	*U*^12^	*U*^13^	*U*^23^
P1	0.0054 (7)	0.0057 (6)	0.0070 (7)	0.0003 (3)	0.0016 (5)	0.0013 (3)
P2	0.0075 (7)	0.0071 (6)	0.0095 (7)	0.0000 (4)	0.0035 (5)	0.0011 (4)
N1	0.0191 (18)	0.0047 (15)	0.0250 (19)	0.0010 (12)	0.0142 (16)	−0.0018 (12)
O1	0.0191 (18)	0.0047 (15)	0.0250 (19)	0.0010 (12)	0.0142 (16)	−0.0018 (12)
N2	0.013 (2)	0.006 (2)	0.016 (2)	−0.0007 (16)	0.008 (2)	0.0032 (17)
O2	0.013 (2)	0.006 (2)	0.016 (2)	−0.0007 (16)	0.008 (2)	0.0032 (17)
N3	0.028 (2)	0.0177 (16)	0.0062 (18)	−0.0054 (14)	0.0048 (16)	0.0015 (13)
O3	0.028 (2)	0.0177 (16)	0.0062 (18)	−0.0054 (14)	0.0048 (16)	0.0015 (13)
N4	0.008 (2)	0.009 (2)	0.013 (2)	0.000	0.003 (2)	0.000
O4	0.008 (2)	0.009 (2)	0.013 (2)	0.000	0.003 (2)	0.000
N5	0.0034 (17)	0.0255 (18)	0.0213 (19)	0.0014 (13)	0.0020 (15)	−0.0079 (15)
O5	0.0034 (17)	0.0255 (18)	0.0213 (19)	0.0014 (13)	0.0020 (15)	−0.0079 (15)

### (coe-PON) Phosphorus oxonitride Geometric parameters (Å, º)

**Table:**

P1—O3^i^	1.568 (3)	P2—O5^iii^	1.584 (3)
P1—N3^i^	1.568 (3)	P2—N5^iii^	1.584 (3)
P1—O1^ii^	1.573 (3)	P2—N1	1.588 (3)
P1—N1^ii^	1.573 (3)	N1—P1^iv^	1.574 (3)
P1—N5	1.574 (3)	N2—P2^v^	1.5530 (9)
P1—N4	1.5755 (17)	N3—P1^i^	1.568 (3)
P2—N2	1.5530 (9)	N4—P1^vi^	1.5755 (17)
P2—N3	1.562 (3)	N5—P2^vii^	1.584 (3)
			
O3^i^—P1—N3^i^	0.0	N2—P2—N3	110.10 (13)
O3^i^—P1—O1^ii^	109.55 (17)	N2—P2—O5^iii^	109.69 (13)
N3^i^—P1—O1^ii^	109.55 (17)	N3—P2—O5^iii^	112.0 (2)
O3^i^—P1—N1^ii^	109.55 (17)	N2—P2—N5^iii^	109.69 (13)
N3^i^—P1—N1^ii^	109.55 (17)	N3—P2—N5^iii^	112.0 (2)
O1^ii^—P1—N1^ii^	0.0	O5^iii^—P2—N5^iii^	0.0
O3^i^—P1—N5	109.7 (2)	N2—P2—N1	108.03 (12)
N3^i^—P1—N5	109.7 (2)	N3—P2—N1	110.13 (18)
O1^ii^—P1—N5	111.04 (17)	O5^iii^—P2—N1	106.79 (19)
N1^ii^—P1—N5	111.04 (17)	N5^iii^—P2—N1	106.79 (19)
O3^i^—P1—N4	107.83 (16)	P1^iv^—N1—P2	135.5 (2)
N3^i^—P1—N4	107.83 (16)	P2—N2—P2^v^	180.0
O1^ii^—P1—N4	111.09 (19)	P2—N3—P1^i^	148.5 (2)
N1^ii^—P1—N4	111.09 (19)	P1^vi^—N4—P1	141.3 (3)
N5—P1—N4	107.57 (15)	P1—N5—P2^vii^	141.3 (2)

Symmetry codes: (i) −*x*+1/2, −*y*+1/2, −*z*; (ii) −*x*+1/2, *y*−1/2, −*z*+1/2; (iii) *x*+1/2, −*y*+1/2, *z*+1/2; (iv) −*x*+1/2, *y*+1/2, −*z*+1/2; (v) −*x*+1/2, −*y*+1/2, −*z*+1; (vi) −*x*+1, *y*, −*z*+1/2; (vii) *x*−1/2, −*y*+1/2, *z*−1/2.

## References

[bb1] Agilent (2014). *CrysAlis PRO*. Agilent Technologies, Yarnton, England.

[bb2] Angel, R. J., Shaw, C. S. J. & Gibbs, G. V. (2003). *Phys. Chem. Miner.* **30**, 167–176.

[bb3] Baumann, D., Niklaus, R. & Schnick, W. (2015). *Angew. Chem. Int. Ed.* **54**, 4388–4391. 10.1002/anie.201410526 25664636

[bb4] Baumann, D., Sedlmaier, S. J. & Schnick, W. (2012). *Angew. Chem. Int. Ed.* **51**, 4707–4709. 10.1002/anie.201200811 22473567

[bb5] Brandenburg, K. (2006). *DIAMOND*. Crystal Impact GbR, Bonn, Germany.

[bb6] Chateau, C., Haines, J., Léger, J. M., Lesauze, A. & Marchand, R. (1999). *Am. Mineral.* **84**, 207–210.

[bb7] Huminicki, D. M. C. & Hawthorne, F. C. (2002). *Rev. Mineral. Geochem.* **48**, 123–253.

[bb8] Léger, J.-M., Haines, J., de Oliveira, L. S., Chateau, C., Le Sauze, A., Marchand, R. & Hull, S. (1999). *J. Phys. Chem. Solids*, **60**, 145–152.

[bb9] Léger, J. M., Haines, J., Chateau, C., Bocquillon, G., Schmidt, M. W., Hull, S., Gorelli, F., Lesauze, A. & Marchand, R. (2001). *Phys. Chem. Miner.* **28**, 388–398.

[bb10] Sheldrick, G. M. (2008). *Acta Cryst.* A**64**, 112–122. 10.1107/S0108767307043930 18156677

[bb11] Sheldrick, G. M. (2015). *Acta Cryst.* C**71**, 3–8.

[bb12] Westrip, S. P. (2010). *J. Appl. Cryst.* **43**, 920–925.

